# A Case of Juxtafoveal Microaneurysm in a Setting of PAMM: Diagnostic and Therapeutic Challenges

**DOI:** 10.1155/crop/7498884

**Published:** 2026-05-07

**Authors:** Parvin Aghayeva, Peter Y. Chang

**Affiliations:** ^1^ Massachusetts Eye Research and Surgery Institution, Waltham, Massachusetts, USA, mersi.us; ^2^ Ocular Immunology and Uveitis Foundation, Waltham, Massachusetts, USA, uveitis.org

**Keywords:** juxtafoveal microaneurysm, migraine, nitroglycerin, PAMM, scotoma

## Abstract

**Purpose:**

We are aimed at reporting a rare case of juxtafoveal microaneurysm in a young adult in the setting of paracentral acute middle maculopathy (PAMM) and discussing potential underlying mechanisms and therapeutic considerations.

**Methods:**

This study describes a single case report and a treatment trial with nitroglycerin.

**Results:**

We present a case of a 38‐year‐old male with recurrent migraines and juxtafoveal microaneurysm who was empirically treated with 0.4‐mg nitroglycerin, resulting in subjective visual improvement and resolution of the lesion on SD‐OCT.

**Conclusions:**

This report highlights the potential role of nitroglycerin as a therapeutic option for recurrent PAMM, emphasizing the need for further investigation into its efficacy and safety.

Summary Statement

The purpose of this work is to describe a novel treatment for a patient with juxtafoveal microaneurysm in the setting of paracentral acute middle maculopathy (PAMM) through a single case report. The case presents a young male patient with recurrent microscotomas for over 2 years, whose complaints stabilized after nitroglycerin.

## 1. Introduction

PAMM was initially recognized in 2013, characterized by a band‐like hyperreflective area seen on SD‐OCT, located at the level of the inner nuclear layer (INL). This finding aligns with a paracentral region of deep retinal whitening observed during ophthalmoscopy or color fundus photography [1].

## 2. Case Presentation

A 38‐year‐old male with a history of longstanding migraines with visual aura first presented to us in 2022 with a persistent temporal scotoma in his right eye. He described the scotoma as resembling two small, closely grouped afterimages or oil stains, without any color. It remained fixed in his visual field, particularly noticeable against bright light and even with his eyes closed. He noted that this was distinct from the floaters he had experienced throughout his life.

**Figure 1:**
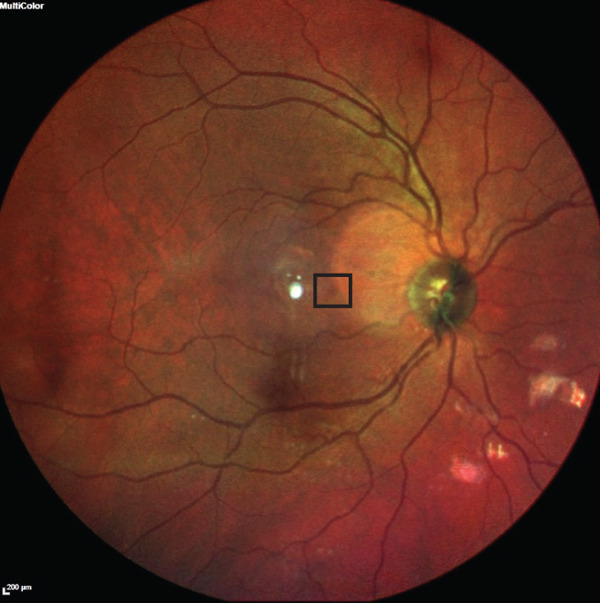
Fundus photo of the right eye (OD) shows lesion (possible microaneurysm) superonasal to the fovea (black square).

**Figure 2:**
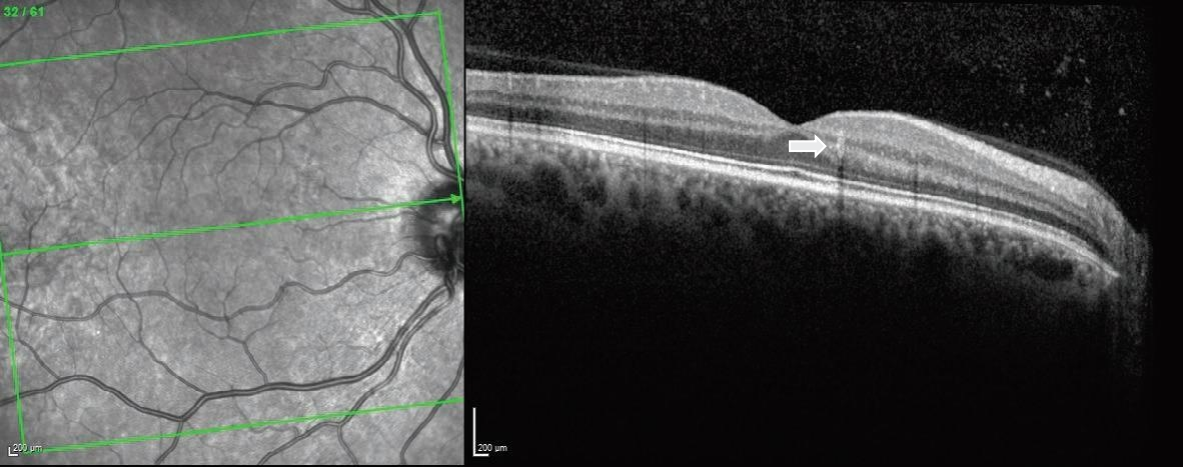
SD‐OCT finding of hyperreflective, band‐like lesion extending from the OPL to the INL, right eye (white arrow).

On presentation, the patient′s visual acuity was 20/20 in the right eye (OD) and 20/15 in the left eye (OS). Anterior segment examination was unremarkable, but dilated fundus examination OD revealed a microaneurysm corresponding to his scotoma (Figure 1). SD‐OCT showed a hyperreflective microaneurysm extending from the outer plexiform layer (OPL) to the INL (Figure 2). Prior to this, he experienced an increase in the frequency of migraines and significant stress. Observation was recommended.

**Figure 3:**
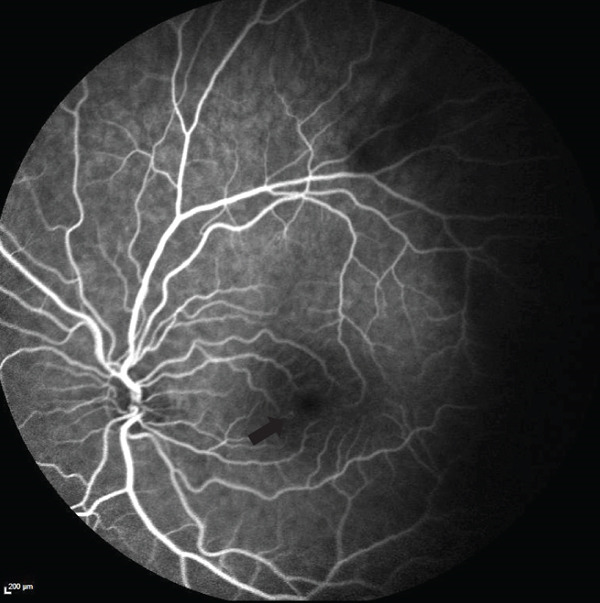
Fluorescein angiography (FA) image of the left eye (OS) shows a focal hyperfluorescent lesion inferonasal to the fovea without associated leakage or staining.

**Figure 4:**
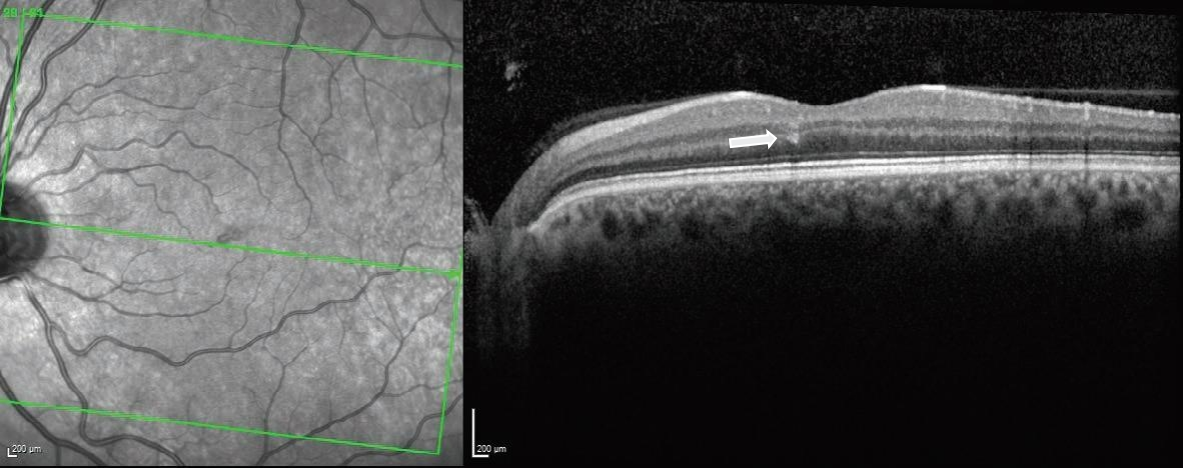
SD‐OCT of the left eye (OS) shows a hyperreflective, band‐like lesion (white arrow).

In early 2023, he presented again with a new scotoma. Fluorescein angiography (FA) revealed microaneurysms near the nasal fovea in OS without leakage (Figure 3), and SD‐OCT showed a new hyperreflective band‐like lesion in the left eye (OS), nasal to the fovea, corresponding to the shape of his visual disturbance (Figure 4). Following this episode, he developed two additional microscotomas in the right eye (OD). He also noted that the scotoma was worsening with caffeine consumption.

The patient independently began taking over‐the‐counter niacinamide 500 mg after conducting his own research, having found evidence that it might help. He reported that this seemed to have already reduced the intensity of his scotomas. Based on this, we considered the potential use of nitroglycerin with consultation with his cardiologist. Given a single case report suggesting efficacy [2], we prescribed nitroglycerin 0.4 mg to be used as needed at the onset of his scotoma.

**Figure 5:**
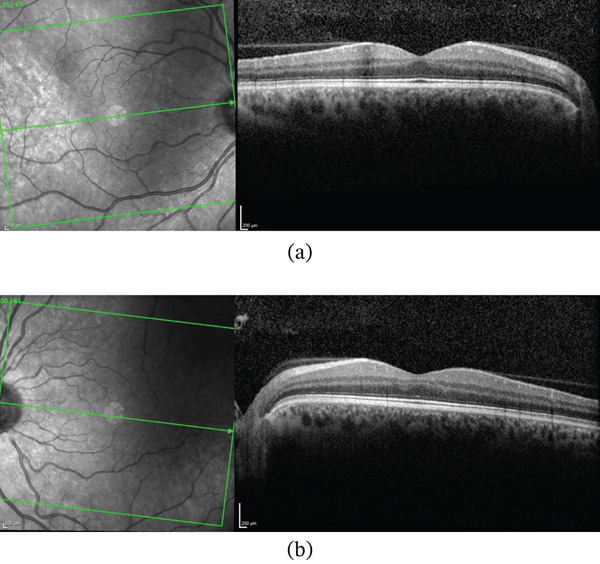
SD‐OCT of (a) the right eye and (b) the left eye shows no lesion corresponding to microaneurysm and PAMM, respectively.

At his 6‐month follow‐up, the patient reported significant improvement in his vision in both eyes (OU) since starting treatment, with no recurrence of flashes or new scotomas. SD‐OCT showed a flat macula in both eyes, with good foveal contours, and the previously seen band‐like gray lesions had resolved (Figure 5). By anticipating the onset of his symptoms, the patient takes 0.4‐mg nitroglycerin which presents a full‐blown scotoma.

**Figure 6:**
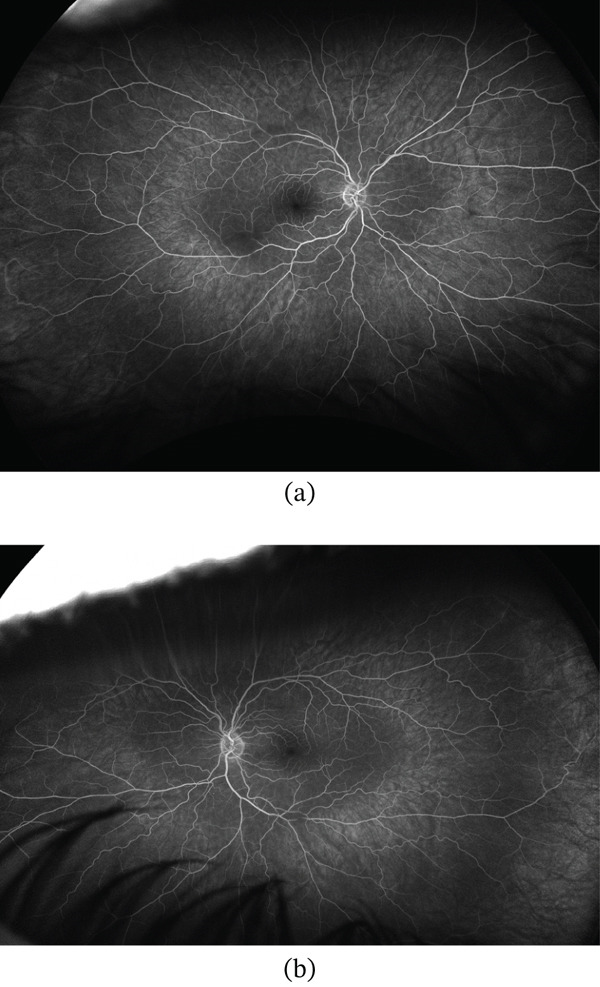
Fluorescein angiography (FA) image of (a) the right eye and (b) the left eye at 12‐month follow‐up shows no staining or leakage.

**Figure 7:**
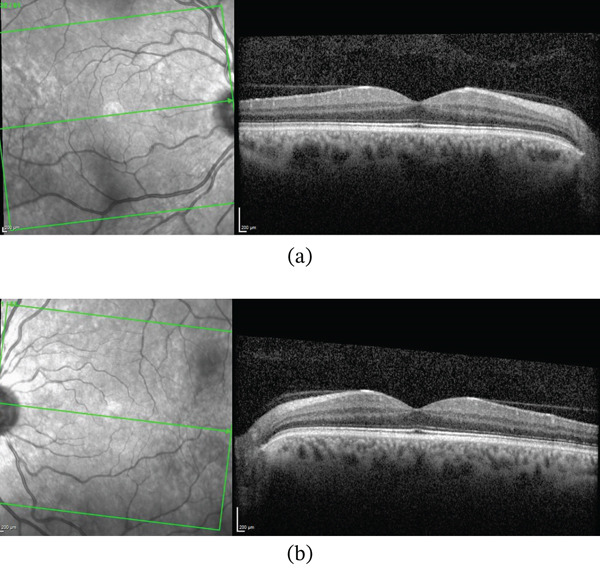
SD‐OCT of (a) the right eye and (b) the left eye at 12‐month follow‐up shows stable retinal architecture.

At 12‐month follow up, repeat FA demonstrated stable retinal perfusion without new areas of staining or leakage (Figure 6). SD‐OCT at the same visit showed resolution of PAMM‐related changes (Figure 7).

## 3. Discussion

PAMM is characterized by macular lesions with notable changes primarily in the INL, as observed on SD‐OCT. PAMM is thought to result from ischemia affecting the deep capillary plexus, which supplies the middle layers of the retina [3].

Research shows that patients with PAMM often present with a newly developed paracentral scotoma, while maintaining 20/20 visual acuity. The lesions, distinct from cotton‐wool spots, may appear as faint gray‐white spots or wedges near the fovea. Unlike cotton‐wool spots, PAMM lesions are deeper within the retina, duller in appearance, and do not follow the nerve fiber layer. These lesions can sometimes resolve before a clinical exam, but SD‐OCT can still detect characteristic hyperreflective bands. As the lesions heal, they typically lead to thinning of the INL, which results in permanent paracentral scotomata.

PAMM is predominantly associated with conditions that lead to retinal ischemia and frequently occurs in conjunction with retinal vascular occlusions. It has been reported in various retinal vascular disorders, including central retinal vein occlusion (CRVO), retinal artery occlusion (RAO), diabetic and hypertensive retinopathy, sickle cell retinopathy, and Purtscher′s retinopathy. Additionally, ocular ischemia resulting from eye compression injuries and inflammatory occlusive retinal vasculitis can also cause these lesions [1, 4, 5]. Several studies have documented PAMM following Covid‐19 infection or vaccination, making it important to investigate potential systemic or external vascular risk factors when PAMM is identified on OCT. Furthermore, PAMM can occasionally develop in otherwise healthy young patients suffering from migraines [4]. Although the association between migraines and PAMM is not well studied, migraine is a complex neurovascular disorder that may contribute to retinal ischemia through several mechanisms. Studies show that trigeminovascular system activates during migraine attack and neurons secrete vasoactive substances resulting in vascular dysregulation and alteration in blood flow [6]. These processes may affect ocular perfusion and contribute to ischemic injury. Chang et al. demonstrated decreased superior peripapillary vessel density in migraine patients with aura compared with patients without aura and healthy controls [7]. Ulusoy et al. found that superficial and deeper retinal foveal vessel densities were significantly less in migraine patients with and without aura than controls on macular OCTAs [8].

Our patient had a history of migraines. At first visit, he reported experiencing an increase in frequency of migraine episodes with visual auras leading up to his presentation. During follow‐up, it became evident that his symptoms worsened with caffeine consumption. Both migraines and caffeine consumption as discussed above could potentially cause PAMM [3, 4].

As it is crucial to perform a thorough evaluation for underlying vascular conditions in patients presenting with PAMM, we conducted a comprehensive systemic work‐up, including laboratory tests such as a lipid panel, A1C, and hypercoagulability screening. The results showed only mildly elevated cholesterol levels. All other measurements, including A1C and hypercoagulability tests, were within normal limits.

Accordingly, there is no known treatment modality for PAMM, especially for patients with no underlying diseases. There is only one anecdotal case describing the use of nitroglycerin in a patient with PAMM [2]. Our report is aimed to further supporting this approach while acknowledging its limitations.

Nitroglycerin acts as a nitric oxide (NO) donor, leading to the relaxation of vascular smooth muscles. There are conflicting studies regarding the effects of NO on retinal arterioles and capillaries. Several studies indicate that NO donors, which typically induce significant dilation in most arteries, may not have the same effect on retinal arteries [9, 10]. Frayser and Hickman [9] reported only modest retinal arterial dilation (5%–6%) following nitroglycerin administration, accompanied by decreased venous oxygen saturation, suggesting reduced retinal perfusion pressure. They proposed that although vessels in the internal carotid system may dilate more readily in response to NO, the ciliary arteries relax significantly more than the smaller retinal arteries. Sodium nitrite failed to induce relaxation in retinal small arteries but produced significant dilation in ciliary arteries, suggesting limited cGMP‐mediated regulation of retinal arterial tone [11]. Another study concluded that sublingual nitroglycerin had no effect on hypoxia‐induced changes in the diameter of retinal arterioles during rest, exercise‐induced increases in arterial blood pressure, or flicker stimulation in healthy individuals [12].

However, the role of NO in regulating retinal arteriolar diameter may be significant in retinal vascular diseases, such as branch retinal vein occlusion [13]. This study supported the hypothesis that decreased NO production leads to arteriolar constriction, as evidenced by a 73.7*%* ± 4*%* reduction in pre‐retinal NO and a 25.4*%* ± 3.4*%* decrease in arteriole diameter in the affected area 2 h after branch vein occlusion [13]. Kitamura et al. demonstrated that endogenous NO or its analog, such as S‐nitrosothiol, is crucial in mediating the substance P–induced dilation of retinal arterioles [14]. Nitroglycerin has been explored for its potential role in treating various retinal vascular diseases. Some findings have indicated improved outcomes in retinal ischemic conditions, such as RAO and CRVO [15]. The rationale is that by improving blood flow, nitroglycerin might alleviate ischemia and prevent further retinal damage.

Although there is no definitive consensus on the effects of NO and its derivatives on retinal arteries, their role in regulating retinal blood flow can be summarized through three integrated mechanisms. First, basal NO production plays a key role in maintaining resting vascular tone [16–18]. Second, retinal vessels exhibit a COX‐1/cAMP–mediated vasodilatory pathway that regulates acute vascular responses [19]. Third, shear stress stimulates NO release, promoting adaptive hyperemia in response to hypoxia and increased metabolic demand [16, 17].

In contrast, retinal capillaries lack smooth muscle cells and pericytes exhibit a more limited vasodilatory response to NO compared with arteriolar smooth muscle. However, NO′s effects on upstream arterioles indirectly influence capillary perfusion by controlling the pressure and flow entering the capillary beds. The functional hyperemia and hypoxic responses mediated by NO occur predominantly through arteriolar dilation, which then increases capillary blood flow secondarily. During acute ischemia, this regulatory system becomes impaired due to increased production of vascular superoxide, which reduces NO bioavailability and disrupts endothelium‐dependent signaling [20]. This mechanism is particularly relevant, as ischemia of the deep capillary plexus is central to the pathophysiology of PAMM.

Interestingly, although nitroglycerin is known to potentially exacerbate migraines, our patient did not experience an increase in migraine episodes after initiating treatment.

Whole observation suggests that the patient′s visual disturbances were likely influenced by a multifaceted interplay of factors, including dyslipidemia, migraines, stress, and caffeine consumption. This complexity underscores the importance of considering multiple risk factors in managing patients with PAMM.

## 4. Conclusion

Our report supports consideration of use of nitroglycerin for patients with recurrent PAMM. PAMM represents a complex interplay of retinal ischemia and vascular health, with ischemic changes in the deep capillary plexus playing a crucial role in its pathophysiology. As is often the case, our patient findings highlight the multifactorial nature of visual disturbances in patients with PAMM.

Although the effects of NO and its donors, including nitroglycerin, on retinal vasculature are controversial, existing literature supports the vasodilatory effects of NO on retinal perfusion, which are essential for alleviating ischemia. Our patient′s favorable response to nitroglycerin treatment, with no exacerbation of migraine symptoms, suggests that it may provide a therapeutic avenue for managing PAMM and its associated visual disturbances.

Given the evidence that nitroglycerin can improve blood flow in retinal ischemic conditions, it warrants further investigation as a potential treatment for acute and recurrent PAMM. Future studies should focus on larger cohorts to explore the efficacy and safety of nitroglycerin in this context, aimed at establishing clearer guidelines for its use in patients with PAMM. Ultimately, a comprehensive approach that considers individual patient factors and underlying vascular conditions will be crucial in optimizing treatment strategies for this challenging condition.

## Author Contributions

**Parvin Aghayeva**: data curation, investigation, and writing—original draft. **Peter Y. Chang**: conceptualization, supervision, and writing—review and editing.

## Funding

No funding was received for this manuscript.

## Disclosure

The authors declare that no third‐party services or individuals contributed to the research or manuscript preparation. None of the entities listed in the financial disclosures above had any role in influencing or directing the research in this study. This work has not been presented at any meetings.

## Consent

Written informed consent was obtained from the patient for publication of this case report and any accompanying images. A copy of the consent form is available for review by the editor upon request.

## Conflicts of Interest

**Peter Y. Chang, MD, FACS**—co‐owner of MERSI; speaker honoraria from Bausch & Lomb, Alimera Sciences, Alcon, and Mallinckrodt Pharmaceuticals; independent contractor with Alimera, Mallinckrodt Pharmaceuticals, and Genentech; Data and Safety Monitoring Board Chair for Ocugen. **Parvin Aghayeva, MD**—no conflicts of interest to report.

## Data Availability

The data that support the findings of this study are available on request from the corresponding author. The data are not publicly available due to privacy or ethical restrictions.
